# Is there benefit in optimising heart failure treatment in over-80 year-old patients? (HF-80 study): study protocol for a randomized controlled trial

**DOI:** 10.1186/1745-6215-13-25

**Published:** 2012-03-06

**Authors:** Romain Eschalier, Frédéric Jean, Bruno Pereira, Séverine Monzy, Charles Vorilhon, Valérie Mactoux, Bernard Citron, Vincent Sapin, Pascal Motreff, Jean R Lusson

**Affiliations:** 1 CHU Clermont-Ferrand, Service Cardiology, F-63000 Clermont-Ferrand; 2 CHU Clermont-Ferrand, Clinical research and innovation direction, F-63000 Clermont-Ferrand; 3 University of Auvergne, F-6300 Clermont-Ferrand; 4 Clermont Université, Université d'Auvergne, UMR6284, F-63000 Clermont-Ferrand; 5 CHU Clermont-Ferrand, Service Biochimy, F-63000 Clermont-Ferrand; 6 CHU Clermont-Ferrand, Service Geriatric, F-63000 Clermont-Ferrand

**Keywords:** Heart failure, Aged and over 80, clinical trial, quality of life

## Abstract

**Background:**

An aging population and better management of various heart diseases explain the exponential growth in incidence and prevalence of chronic heart failure, with poor prognosis and heavy health costs. Medical management is codified in international guidelines. The management of heart failure in over-80 year-old patients follows these guidelines, but no clinical trials have been able to confirm benefit. Moreover, registries show down-prescription of heart failure treatments in the elderly and over-80s.

**Methods/Design:**

We present the design of the HF-80 ("Is there benefit in optimising heart failure treatment in over-80 year-old patients?") study, which is a prospective randomised open-label clinical trial with blinded end-points, designed to evaluate the effect of optimising management by adhering to guidelines in over-80 year-old heart failure patients. Patients over 80 years of age admitted with acute heart failure will be included. The primary endpoint is to assess quality of life at 6 months on the Minnesota questionnaire. The secondary endpoints are to assess the effect of optimised management on quality of life, mortality, readmission for acute heart failure, cardiac fibrosis and economic data at 12 months. 80 patients will be included, divided into 2 groups: group A, with usual heart failure management by general practitioners; and group B, with optimised management based on international guidelines.

**Discussion:**

It is necessary to assess the benefit of guidelines in over-80 year-old heart failure patients because of the fragility of this population and the elevated risk of iatrogenic complications.

**Trial Registration:**

Clinical trials.gov number: NCT01437371.

## Background

An aging population and improved management of various heart diseases involving ischemic aetiologies explain the growth in incidence and prevalence of chronic heart failure (HF) [1], with high complication rates [2,3] and heavy costs (more than 1% of total health care costs in industrialised countries). Mean age at diagnosis of HF was 70 years in the Framingham cohort [3]. Incidence and prevalence increase exponentially with age [3].

Angiotensin-converting enzyme inhibitors (ACEi) [4,5] beta-blockers [6,7], mineralocorticoid receptor antagonists [8], and angiotensin receptor blockers [9,10] provide first-line therapeutic management of HF as recommended in international guidelines [11,12]. These, however, are based on studies conducted on younger patients (mean age between 61 and 71 years: Table 1). Clinical management of over-80 year-old HF patients conforms to these guidelines, but no clinical trials have been able to confirm their benefit in this population. Comorbidity and iatrogenic complications may impair the effect of common treatments. Recent clinical trials in HF management continued to recruit younger subjects, because of the need to highlight a beneficial effect of the treatment tested [13-15]. Some studies have reported benefit with such treatments in this kind of population [16,17]. Registries and observational studies highlighted down-prescription, especially for ACEi and beta-blockers, in elderly and over-80 year-old patients [18,19].

**Table 1:** Mean age of main studies in HF management

Clinical studies	Ages of placebo Group (years old)	Ages of studied drug group (years old)
*HF subgroup of Hyvet (hypertension study)*	83.5 ± 3.1	83.6 ± 3.2

*Seniors*	76.1 ± 4.8	76.1 ± 4.8

*Charm-Alternative*	66.8 ± 10.5	66.3 ± 11

*Merit HF*	63.7	63.9

*Charm-Added*	64.1 ± 11.3	64.0 ± 10.7

*Consensus*		71

*Solvd*	59.1	59.1

*Cibis II*	61	61

*Emphasis-HF*	68.6 ± 7.6	68.7 ± 7.7

*Ephesus*	66 ± 12	64 ± 11

*Rales*	65 ± 12	65 ± 12

*Atlas study*	Low-dose of lisinopril: 63.6 ± 10.3	High-dose of lisinopril: 63.6 ± 10.5

*Elite-1 trial*	Losartan: 74(5-8)	Captopril: 73 (6-1)

*Elite-II trial*	Losartan: 71.4 (6-7)	Captopril: 71 (6-9)

*Shift*	60.1 ± 11.5	60.7 ± 11.2

*Copernicus*	63.4 ± 11.5	63.2 ± 11.4

*Valiant*	Captopril: 64.9 ± 11.8 Valsartan: 65 ± 11.8	Valsartan + Captopril: 64.6 ± 11.9

*Dig*	63.9 ± 11.7	63.8 ± 11

## Methods/Design

Heart failure management is now clearly codified for the general population, thanks to several studies and guidelines. Management of over-80 year-old HF patients, however, is simply extrapolated from their findings. No study has specifically assessed optimised HF treatment in the over-80s, where potential benefit could be counterbalanced by co-morbidity and iatrogenic complications. The HF-80 clinical trial was designed as a pilot study to investigate whether optimised management has an effect on quality of life (QOL) in over-80 year-old HF patients.

### The HF-80 Study

#### Objectives and endpoints

The primary objective of this pilot study is to assess optimised HF management, according to the guidelines of the European Society of Cardiology (ESC) [11,12], in terms of impact on QOL in over-80 year-old patients at 6 months.

The secondary objectives are to evaluate the effect of optimised management on:

• Quality of life at 12 months

• Mortality at 12 months

• Readmission for acute HF at 12 months

• Cardiovascular events at 12 months

• Cardiac fibrosis, evaluated by collagen peptides.

The primary assessment criterion is QOL at 6 months, assessed on the Minnesota Living with Heart Failure Questionnaire (LHFQ) [20].

The secondary assessment criteria are QOL at 12 months, measured by both the SF 12 [21] and LHFQ [20] (to check which scale is most suited to this population), mortality at 12 months, number of readmissions for acute HF, cardiovascular events at 12 months, evolution in New York Hospital Association (NYHA) class (at baseline, 6 months and 12 months), and evolution in 6-minutes walking test (6MWT) (at baseline, 6 months and 12 months). Finally, an analysis of the medical and economic interest of this support will be conducted.

This pilot study will also help lay the groundwork for a French national multicentre study of the management of octogenarian HF patients to find what support is most appropriate.

#### Study design

The HF-80 pilot study is a prospective randomised single-centre open-label clinical trial with blinded endpoints. Current lack of knowledge regarding management for this population requires a pilot study, to harvest data before starting a clinical trial to assess mortality. The study population is over-80 year-old patients recently admitted for acute HF. Inclusion and exclusion criteria are listed in Table 2.

**Table 2:** Inclusion and exclusion criteria in HF 80 study

Inclusion criteria:	
	Aged over-80 year-old subjects
	Hospitalized for an acute heart failure
	Left Ventricle Ejection Fraction ≤ 35%
	Evaluated life expectancy (Seattle Heart failure score) > 1 year
Exclusion criteria:	
	Dementia (evaluated by MMSE)
	Do not understand French language
	Followed with an optimized management
	With reduced mobility
	Recruited in another clinical trial or in a HF management network
	Acute HF with curable aetiology: cardiovascular surgery for coronary artery bypass graft or valvular replacement, angioplasty
	MDRD < 30 ml/min/1.73 m^2^

Dementia will be assessed by the Mini mental state examination (MMSE): patients with scores under 24/30 will not be included. For the very elderly, a preliminary walking test will be performed, to exclude patients with reduced mobility, who are moreover very often subject to dementia.

#### Randomisation

Included patients will be randomly allocated to either arm in a 1:1 ratio. The randomisation list is generated by minimisation [22] to maintain better balance than with traditional block randomisation. Stratification will be performed on gender (female/male), readmission (yes/no) and residence (at home/not at home). When a patient is considered eligible and informed consent has been obtained, randomisation will be performed automatically (on software) by an independent statistician.

#### Investigation Procedure

**Figure 1:**
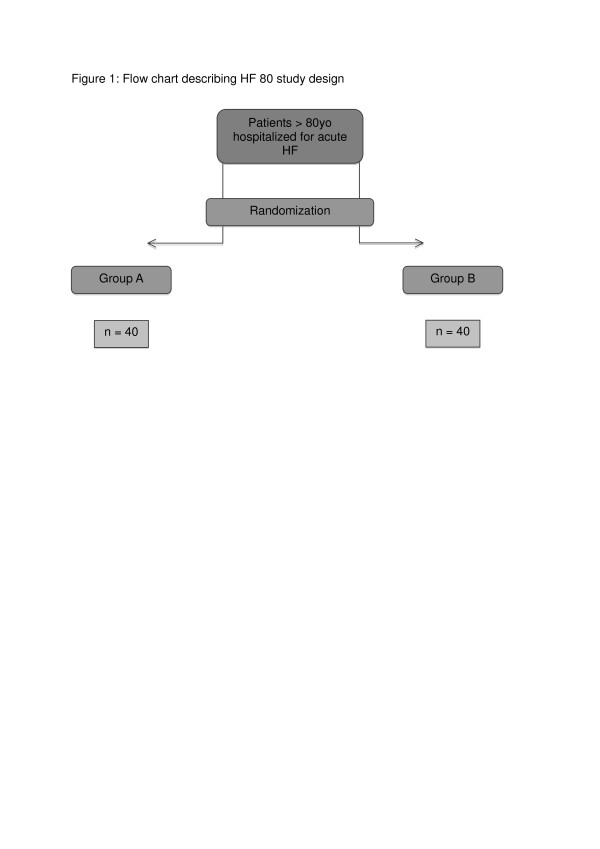
**Flow chart describing HF 80 study design**. HF: heart failure.

Over-80 year-old patients will be recruited and randomised into 2 groups of 40 (Figure 1):

• Group A: "usual care" management by the patient's usual general practitioner (GP) and cardiologist;

• Group B: "optimised" management strictly adhering to ESC guidelines [11,12], performed by the patient's usual GP and cardiologist plus day-hospital.

Patients will be included in HF-80 at discharge from acute HF care. Before inclusion, trans-thoracic echocardiography will confirm that left ventricle ejection fraction is under 35% and that no exclusion criteria are present. During hospital care, patients of both groups will receive the same treatments, so far as possible using all HF therapeutics; different hospital treatment according to group would not be ethically acceptable. Mean hospital stay is about 10 days, which is too short to induce bias, as medical management optimisation applies only after discharge. Optimised management will be assessed with long-term prognosis.

**Figure 2:**
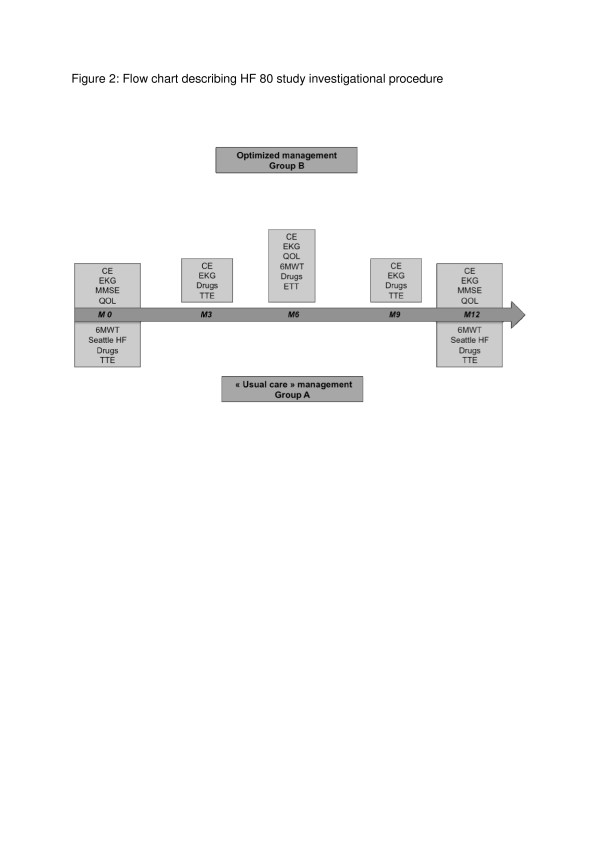
**Flow chart describing HF 80 study investigational procedure**. CE: clinical examination; EKG: electrocardiogram; MMSE: mini mental state examination; QOL: quality of life scale; 6MWT: 6 minutes walking test; Seattle HF: Seattle heart failure score; TTE: transthoracic echocardiography.

An identical assessment is to be performed at baseline and at 12 months for all patients (groups A and B) (Figure 2):

• Clinical examination: NYHA class; weight; height; heart rate; blood pressure; right, left or both HF symptoms; triggering factor; cardiovascular risk factors; thyroid pathologies; personal cardiovascular history;

• Quality of life questionnaires (LHFQ [20], SF 12 [21]);

• Six-minutes walking test (6MWT);

• Electrocardiogram (EKG);

• Transthoracic echocardiography (TTE);

• Blood test (haemoglobin, kaliaemia, natraemia, liver assessment, bilirubin, Modification in Diet Renal Disease (MDRD) [23], N-Terminal of pro-Brain Natriuretic Peptide (NT-pro-BNP), and nutritional tests (C-reactive protein, albumin, prealbumin and orosomucoid);

• Renin, aldosterone and collagen peptides [type 1 collagen telopeptide (ICTP), aminoterminal propeptide of type I procollagen (PINP), aminoterminal propeptide of type III procollagen (PIIINP)], and galectin 3;

• Mini mental state examination (MMSE) [24].

During primary hospital care for acute HF and at discharge, both groups will have the same management with initiation of the same treatments, following guidelines [11,12].

Group A subjects will be followed up by their usual GP and cardiologist without any day-hospital care ("usual care" management). The GP and cardiologist will manage treatment optimisation as they are used to doing.

Group B subjects will have the same management as group A but will also receive day-hospital care. The first 3 visits will be at 3 weekly intervals, except in case of unstable HF, and then every 6 weeks for 12 months, to optimise treatment.

During each day-hospital visit in group B, certain data will be recorded to optimise HF management:

• Clinical examination (NYHA class, HF symptoms, blood pressure, heart rate);

• Blood sample (kaliaemia, MDRD [23], NT-pro-BNP);

• Electrocardiogram;

• Optimised treatment doses, if possible, for ACEi, beta-blockers, angiotensin receptor blocker and mineralocorticoid receptor antagonist.

All the visits and contents are described in Table 3.

**Table 3:** Schedule of visits and contents

	Group A		Group B					
	***Enrollement hospitalization***	***Month 12 visit***	***Enrollement hospitalization***	***Day hospital visits***	***Month 3 visit***	***Month 6 visit***	***Month 9 visit***	***Month 12 visit***

QOL questionnaires (LVHQ, SF12)	X	X	X			X		X

Clinical examination (BP, HR, weight, NYHA)	X	X	X	X	X		X	X

EKG	X	X	X	X	X	X	X	X

6MWT	X	X	X					X

Na, Hb, SGOT, SGPT, Bili	X	X	X					X
K, MDRD, NT-pro-BNP	X	X	X	X	X	X	X	X
PCR, oroso, alb, prealb	X	X	X					X

TTE	X	X	X		X	X	X	X

Blood laboratory	X	X	X		X	X	X	X

Treatments	X	X	X	X	X	X	X	X

BP: blood pressure; HR: heart rate; EKG: electrocardiogram; MMSE: mini mental state examination; QOL: quality of life; 6MWT: 6 minutes walking test; Seattle HF: Seattle heart failure score; TTE: transthoracic echocardiography; CRP: C reactive protein; oroso: orosomucoid; alb: albumin; prealb: prealbumin; MDRD: Modification in Diet Renal Disease; NT-ProBNP: N terminal pro brain natriuretic peptid; K: kaliemia; Na: sodium blood concentration; Hb: hemoglobin; bili: biliubin; SGPT: Serum Glutamopyruvate Transferase, SGOT: Serum Glutamooxaloacetate Transferase.

Patients in both groups will have a notebook, for 12 months. GPs and cardiologists will note all changes in treatment and management.

#### Transthoracic echocardiography analysis

TTE will be performed using a 2.5 MHz probe (VIVID 9, General Electrics), by a single operator [25], at inclusion and at 12 months, in both groups. TTE will also be performed at 3, 6 and 9 months for group B in day hospital, and according to "usual management" criteria for group A.

The following measurements will be taken in each subject:

2D/MM modes: Right ventricle (RV) diameter (mm); interventricular septum thickness at end diastole and end systole (mm); left ventricle (LV) posterior wall thickness at end diastole and end systole (mm); LV diameter at end diastole and end systole (mm); LV volumes at end diastole and end systole (ml); LV ejection fraction measured by the biplane Simpson method (%); LV mass index (g/m2); inferior vena cava diameter (mm); left and right atrium areas (cm^2^); RV volume; RV ejection fraction; TEI myocardial performance index; and tricuspid annular plane systolic excursion (TAPSE).

Doppler modes: transmitral flow, including peak E wave (cm/s), peak A wave (cm/s), E/A ratio, E-wave deceleration time (m/s); pulmonary venous waveforms, including peak systolic (S) velocity, peak anterograde diastolic (D) velocity, and S/D ratio; VP, Color M-Mode flow propagation velocity; relaxation isovolumic time (m/s); cardiac output, tricuspid E wave; tricuspid regurgitation (none/mean/moderate/severe); systolic pulmonary arterial pressure; mitral regurgitation (none/mean/moderate/severe); and dp/dt for left and right ventricle.

Doppler tissue imaging mode: lateral and septal E' wave and A wave at mitral annulus (cm/s), lateral and septal S wave at mitral annulus (cm/s); S' and E' wave at tricuspid annulus (cm/s).

2D-Strain mode: Loops obtained by 2D on 3 cycles, with apical 4-cavities, 3-cavities, 2-cavities and parasternal short axis views, with frame rate > 60 frames/s.

#### 6-minutes walking test

This will be performed for each patient at inclusion and at 12 months, and also at 6 months for group B [26]. The test will be made in hospital on a 60-metre track. The patients will be requested to walk at their tolerance threshold for 6 minutes. Before the beginning of each test, respiratory frequency, heart rate, blood pressure and effort perception on the Borg scale [27,28] will be measured. At the end of each test, the same parameters will be measured again. The final result of the 6MWT is the total distance covered (metres) in 6 minutes.

#### Blood tests

The usual blood tests made during acute HF hospitalisation will be performed by the hospital biochemistry laboratory: haemoglobin, K, Na, liver assessment, bilirubin, MDRD [23], NT-pro-BNP, natriuresis, and nutritional tests (C-reactive protein, albumin, prealbumin and orosomucoid), using the AutomateVista 1500 (Siemens HealthCare Diagnostics)]. Renin (Liaison Direct Renin kit, Diasorin), aldosterone (ELISA Kit (E90911Hu), USC N Life Science Inc.), PIIINP (ELISA Kit (E90573Hu), USC N Life Science Inc.), PINP (ELISA Kit (E90957Hu), USC N Life Science Inc.), ICTP (ELISA Kit (E90665Hu), USC N Life Science Inc.) and galectin 3 (ELISA kit (DGAL30), R and D systems) will be analysed at end of trial, after storage at -80°C.

#### QOL scales

It was decided to implement two QOL questionnaires (LHFQ [20] and SF 12[21]) to assess which is more adapted to over-80 year-old HF patients. Both are well-validated and widely used. The SF12 is used on a daily basis in the geriatric department of our hospital and maybe more adapted to the elderly. A second endpoint is to assess the efficiency of this shorter QOL questionnaire, SF12, as compared to the LHFQ, in a very elderly population. They will be completed at inclusion and at 12 month for each subject. QOL scales will be sent to all participants (group A and B) at 6 months (before the 6 month visit for group B) and will be filled in by the patients without any help from medics or paramedics (to avoid bias).

### Statistical Considerations

Due to lack of information in the literature concerning the management of over-80 year-old HF patients, it is difficult to estimate correct sample size. The number of subjects to be included is extrapolated from data obtained on younger patients, according to which 40 subjects per group will be included.

With a 2-tailed significance threshold of 0.05, statistical power of 90% and allowing for 15% loss to follow-up, 80 patients will be needed to show a difference of 20 points (σ = 25) in quality of life (LHFQ) at 6 months between the 2 randomisation arms [29]. Morcillo et al. demonstrated that a 20-point difference in LFHQ is significant and feasible in such a population at 6 months' follow-up. An interim analysis is planned. For 34 evaluable patients (17 per group), a difference in QOL score between the 2 arms will be considered significant for an adjusted α equal to 0.003 (Lan and DeMets, EaSt^© ^software). Termination for futility can thus be considered.

The number of patients included and the curve of the inclusions, the theoretical number of visits for the number of patients included, the number of visits actually made and the ratio will be presented for the 2 groups. The cumulative duration of follow-up and the "total follow-up/expected cumulative follow-up" ratio will be calculated.

All analyses will be performed on an intention-to-treat basis. Clinically relevant baseline variables and primary and secondary endpoints will be compared between groups by Chi^2 ^or Fisher-exact tests (categorical variables) and by Student's t tests or Mann-Whitney tests as appropriate (continuous variables). Multivariate analyses to control for confounding effects of variables will be performed.

The survival analysis will be conducted in univariate analysis by log-rank test to compare survival curves following Kaplan-Meier, and in multivariate analysis by the Cox proportional regression model.

To measure the evolution of parameters over time points (visits), longitudinal data analysis will be conducted by ANOVA for repeated measures followed by Tukey-Kramer post-hoc test and by random effects models to measure within-subject correlation taking account of effects over time (random intercept and slope). The impact of covariates, such as randomisation group, will be explored to assess the impact of strategies on QOL score.

Treatment compliance will be analysed initially in a descriptive step and, if necessary, included in multivariate analysis.

A 2-tailed p value of 0.05 will be considered statistically significant (except in interim analysis). All analyses will be performed by STATAv11 (StataCorp, College Station, Texas, USA).

## Discussion

The aging of the population [1] is increasing hospital admission for acute HF, particularly in subjects over 80 years of age. This is a special population, often with multiple comorbidity and a high risk of iatrogenic complications, in whom it is difficult to implement all recommended treatments at optimal doses. There is a significant difference between the optimal treatment doses according to the literature on HF and the doses actually prescribed to inpatients [18,19,30]. Guidelines are extrapolated to this population without knowing whether there is benefit.

Unfortunately, clinical trials on HF have recruited young patients. Clinical studies in cardiology, and particularly in HF, recruit young subjects at the expense of seniors who are underrepresented if not excluded [31-33]. This trend was confirmed by recent large studies of therapeutic drugs (Emphasis-HF [13] and SHIFT [14] subjects were aged, respectively, 68 and 60 years) and electric therapy [15]. The SENIORS study [17] confirmed the value of beta-blockers in patients over 70 years old; benefit, however, was especially pronounced in patients younger than 75 years. The ELITE study [34] was probably the first large HF trial to decide to exclude young patients: only over-65 year-olds were recruited, two-thirds aged 70 or older; the safety of losartan and captopril in HF was demonstrated. These findings were confirmed in morbidity and mortality, but in a younger population (over-60 year-old HF patients [35]. HYVET [16], an antihypertensive clinical trial, included patients over 80 years of age and showed benefit with medical treatment in hypertension in this age group, including in the subgroup with onset of HF (64% reduction). Observational studies and registries show down-prescription of recommended treatments, including ACEi and beta-blockers, in over-80 year-old HF patients [18,19,30]. This requires specific studies in elderly patients (> 80 years old) to consider the respective interest of different classes and recommended therapeutic doses. Given the aging population and the exponential prevalence of over-80 year-old HF patients, it is primordial to know if optimised management with increased treatment doses shows benefit in this population. Iatrogenic complications such as chronic renal failure, orthostatic hypotension or hyperkaliemia could aggravate clinical status in this population and counterbalance expected benefit. The ATLAS study confirmed that intermediate and high-dose lisinopril was more effective in terms of death and hospital admission than low doses, but with side effects (dizziness, hypotension, worsening renal function, hyperkaliaemia), although not such as to lead to termination of lisinopril [36].

A potential bias is present in this design. Patients in the "optimised" group will have more cardiology day-hospital visits; GPs will be in charge of their own management optimisation, as recommended in the ESC guidelines.

Divergent data are present in the literature on elderly HF patients, and there is no consensus on managing over-80 year-old patients. The growing size of this population requires clinical trials to confirm that the HF management validated in younger subjects is also effective in those over 80 years of age.

### Trial Status

HF-80 study is in an activating recruiting phase since October 2011. Institute's committee on human research (CPP Sud Est VI) agreement was obtained in April 2011. Subjects will give their informed consent before being enrolled in the study. AFFSAPS agreement was obtained in May 2011. HF-80 will start enrolling patients in October 2011. Study completion date is estimated at December 2012. Clinical trials.gov number: NCT 01437371.

## List of abbreviations

HF: Heart Failure; QOL: Quality of life; LVHQ: Minnesota living with heart failure questionnaire; LV: Left ventricle; 6MWT: 6 minutes walking test; TTE: transthoracic echocardiography; ESC: European society of cardiology; ACEi: angiotensin conversing enzyme inhibitors; NYHA: New York Heart Association; GP: general practitioner.

## Competing interests

This clinical trial is funded by the support of Laboratoires Servier and Sorin group.

The authors declare that they have no competing interests and no other source of fundings than those cited above.

## Authors' contributions

RE, FJ, SM, BP, and CV participated in the design of the study. RE and FJ participated in writing the manuscript. BP performed statistical section. VS participated in the biological part of the study. PM, JRL, BC helped to draft the manuscript, and participated in study design and coordination. All authors read and approved the final manuscript.
